# Spontaneous cystic biloma after laparoscopic cholecystectomy treated conservatively: A case report

**DOI:** 10.1016/j.amsu.2021.102435

**Published:** 2021-05-31

**Authors:** Adeodatus Yuda Handaya, Aditya Rifqi Fauzi, Joshua Andrew, Ahmad Shafa Hanif, Kevin Radinal, Azriel Farrel Kresna Aditya

**Affiliations:** Digestive Surgery Division, Department of Surgery, Faculty of Medicine, Universitas Gadjah Mada/Dr. Sardjito Hospital, Yogyakarta 55281, Faculty of Medicine, Universitas Gadjah Mada/Dr Sardjito Hospital, Yogyakarta, Indonesia

**Keywords:** Biloma cyst, Post laparoscopic cholecystectomy, Percutaneous drainage

## Abstract

**Introduction:**

Biloma forms due to common bile duct (CBD) injury as a laparoscopic cholecystectomy complication. Spontaneous localized biloma forming cysts in the biliary duct is rare.

**Presentation of case:**

We report a 47-year-old male with complaint of a painful lump in the upper abdomen two months after laparoscopic cholecystectomy. Magnetic resonance cholangiopancreatography (MRCP) found a large epigastric cyst mass, without any signs of CBD injury. Patient was managed with percutaneous drainage in the outpatient clinic and kept the contents of the drainage bag for evaluation. After two months follow-up the outcome was favorable.

**Discussion:**

Biloma forming cysts is a very rare complication post laparoscopic cholecystectomy. Biloma most common occurs as free fluid in the abdominal cavity. Clinical diagnostics, intraoperative historical evaluation and support with MRCP may determine the treatment options. Decision to manage with non-operative procedures by percutaneous drainage and evaluations of the patient in the outpatient clinic had a favorable outcome.

**Conclusion:**

Post laparoscopic biloma cysts are a very rare case. Management with percutaneous drainage in an outpatient clinic and ambulatory drainage is an effective and safe procedure.

## Highlights

• Spontaneous localized biloma forming cysts in the biliary duct is rare.
• Biloma most common occurs as free fluid in the abdominal cavity.
• MRCP can help make the diagnosis.
• Can be managed with percutaneous and ambulatory drainage.

## 1 Introduction

Biloma is a collection of the bile outside the biliary tract within the abdominal cavity most commonly occurring in the subhepatic space. While the exact mechanism is not completely understood, choledocholithiasis is the most common cause of spontaneous biloma and other causes include abdominal trauma and surgery, bile duct tumors, liver infarction, percutaneous catheter drainage, transhepatic cholangiogram, and endoscopic retrograde cholangiopancreatography (ERCP) [1,2].

Bilomas after cholecystectomy are relatively uncommon. The reported incidence is only 0.3%–2%. They usually present with right upper quadrant or epigastric pain, nausea, abdominal distension, fever and leukocytosis. In the initial evaluation, abdominal ultrasound is recommended to identify the type of leak in patients suspected of biliary leak [3,4]. Biloma management may vary from percutaneous catheter drainage to overt surgical treatment. If the leak is small, it can resolve spontaneously in few days [5]. This study reports a biloma cyst or a spontaneous biloma localized cyst forming two months after surgery without injury of the biliary tree which has not previously been reported elsewhere in the literature. This work has been reported in line with the SCARE checklist [6].

## 2 Presentation of case

A 47-year-old patient underwent treatment at the outpatient clinic after presenting with a complaint about a painful lump in the upper middle abdomen (epigastrium quadrant) 2 months after laparoscopic cholecystectomy and the lump getting bigger.

Evaluation history of surgery did not reveal the presence of bile nor bleeding in the postoperative evaluation. At the time of surgery there were doubts about the assessment of the cystic duct and common bile duct (CBD), so we decided to evaluate the CBD with blunt dissection of the visceral peritoneum.

**Fig. 1:**
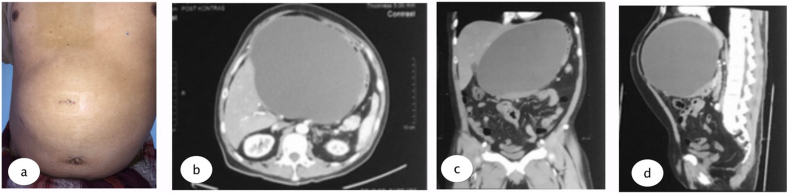
a. Epigastric lump and post laparoscopic scar 2 months after laparoscopic cholecystectomy; b. MSCT axial view, c. Coronal view, and d. Sagittal view show biloma cyst.

**Fig. 2:**
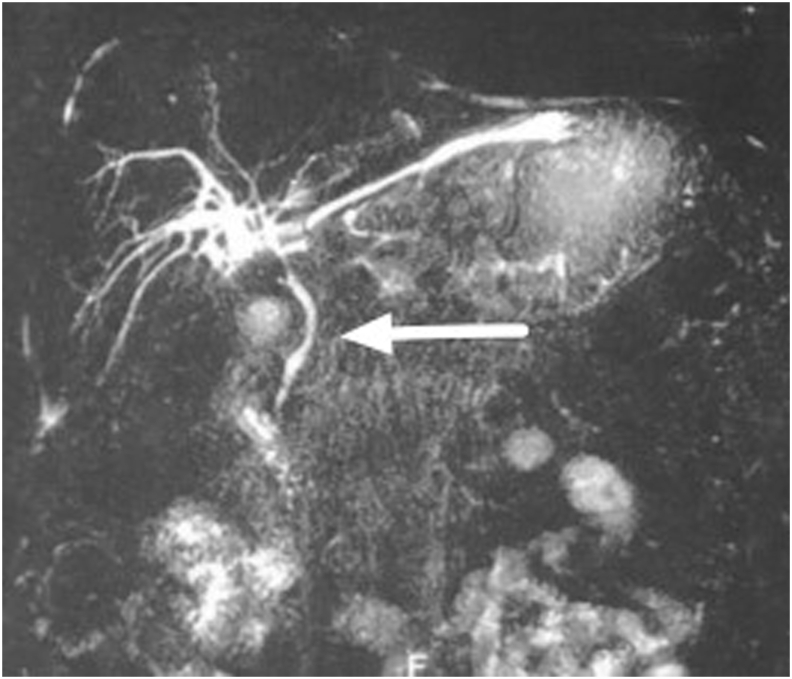
The MRCP revealed intact common bile duct.

**Fig. 3:**
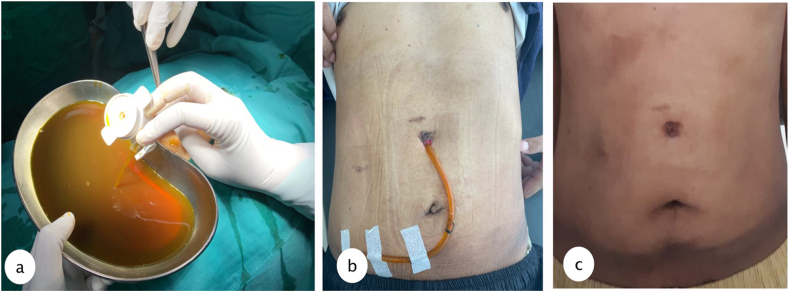
a. Drain pan from the biloma cyst with 5mm laparoscopic trocar, reveal the bile product from cyst. b. insertion of 16 FR NGT (2 weeks after procedure) c. 2 months post drainage, evaluation in outpatient clinic after tube removal.

**Fig. 4:**
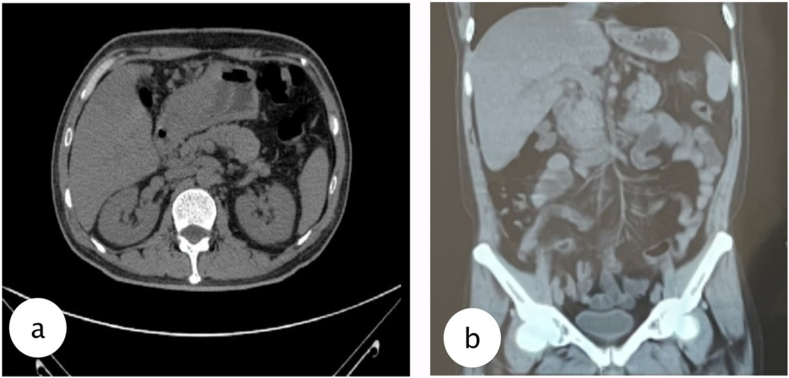
MSCT evaluation after 2 months drainage, a. axial view, b. coronal view shows the biloma was drained.

The physical examination found mild jaundice and a spongy mass in the epigastric area. Laboratory examination was conducted with the results: Hb 15.6, Leukocyte 8.76, Total Bilirubin 1.5, direct bilirubin 0.86, SGOT 103, and SGPT 427. A large epigastric cystic mass, without signs of CBD injury could be seen in the Magnetic Resonance Cholangiopancreatography (MRCP). The patient underwent multi slice computed tomography (MSCT) which revealed a cystic mass in the patient's epigastrium, without discontinuity in CBD (see Fig. 1, Fig. 2, Fig. 3, Fig. 4).

Percutaneous drainage was performed under local anesthesia using 16 FR nasogastric tube (NGT) guided with 5 mm laparoscopic trocar to the most prominent area which could be done in outpatient clinic, and the tube connected to reservoir bag to collect the product. Due to its size we inserted the tube guided from the MSCT results. Evaluation was done every week of the drain product and gradually it reduced after 2 months until the product was negative and the drain could be removed. Results from MSCT evaluation were favorable.

## 3 Discussion

Biloma is an encapsulated collection of bile that can occur outside or inside the biliary system within the abdominal cavity. It is a rare condition with an incidence of 0.3%–2% [1]. Mean time for developing post-cholecystectomy biloma is about 2 weeks from the surgery, but in the literature, cases up to 5 years from the surgery have been reported. In rare cases, biloma can present on postoperative day 5–15 and infrequently, past 1 year [4,7]. In this study, the patient presented with complaints of a painful lump in the upper abdomen and no jaundice two months after laparoscopic cholecystectomy.

Post laparoscopic biloma cysts have not been reported before. Biloma most commonly occurs as free fluid in the abdominal cavity but not collected in a cyst. Biloma as a complication of laparoscopic cholecystectomy can be the result of the leakage from an inadequately secured cystic duct stump of an accessory right hepatic duct, cystic duct, duct of Luschka in the gallbladder fossa of the liver or injury to the main biliary ducts [1,8,9]. We found the very rare case that biloma collected in an isolated cyst at the epigastric area. The risk of injury of CBD increases if the cystic duct has similar size with CBD which can lead to misidentification. In our case, we had doubts about the assessment of the cystic duct and CBD, so we decided to dissect the serous layer of the CBD to ensure the junction of the cystic duct and CBD. This procedure made ischemia and micro perforation to the serous layer of CBD and caused further bile leaking and collecting in the capsulated mass resulting in a rare biloma cyst. Intra operative cholangiography (IOC) was recommended to confirm the structure of the cystic duct and CBD.

Our patient underwent MRCP that found a large epigastric cystic mass full of leaking bile, without any signs of CBD injury. Abdominal imaging, such as abdominal ultrasound (US), multi-slice computed tomography (MSCT), magnetic resonance cholangiopancreatography (MRCP), and Chole-scintigraphy using 99mTc hepatobiliary iminodiacetic acid (HIDA) scan, are crucial to identify biloma and rule out other possible etiologies [10]. Ultrasound (US) is preferable as the first-level imaging method, since it is non-invasive, fast and easy to perform. US findings suggest biloma are a hypo-anechoic fluid collection, with well-defined margins, sometimes encapsulated, mono- or pluriconcamerate appearance, in a typical location (right upper abdominal quadrant: sub- or intrahepatic, below the diaphragm) and no vascularity on color Doppler US. The size of a biloma may vary from a few centimeters up to 40 cm in diameter. But the sensitivity of abdominal US is low (70%), though it is used as the initial imaging in the evaluation of biloma. The sensitivity and specificity of CT scan are approximately 90% and those of MRI are above 95% in the detection of biloma and bile leak; however, smaller bilomas can be missed [4,10]. The MRCP sequences are helpful to identify the source of the biliary leak. Specifically, thin-slab MRCP sequences may show the point of communication between the fluid collection and bile ducts. Thin-slab MRCP sequences are also helpful in depicting the detailed anatomy of the biliary system and in detecting accessory biliary ducts, which could potentially be the source of the bile leak [9,11].

In this report, we performed percutaneous drainage with 5 mm laparoscopic trocar and 16 Fr NGT for drainage with local anesthesia which could be done in the outpatient clinic, and the patient was discharged on the same day with the tube connected to a collecting bag. Observation was done every week to evaluate the drain product, for any sign of infection and to measure size of lump. Treatment of biloma depends on the severity of the disease. Asymptomatic patients with small sized biloma can be managed conservatively. Open surgery is not the initial treatment nowadays, because there are many other treatment options. Open surgery is often chosen for diffuse biloma with persistent leakage or with underlying disease to secure the effectiveness of bile drainage and control intraabdominal infection. Most postoperative bilomas are managed by percutaneous drainage with the placement of stent endoscopically. If the drainage and conservative treatment with broad-spectrum antibiotic therapy fail, the advanced management with stent placement for prolonged drainage, micro-coil, and ethanol intrahepatic embolization would be the treatment options [1,3].

Our patient after 2 months follow-up has no complaints, and when the abdominal lump was gone, then tube could be removed. The patient was evaluated with MSCT showing the biloma was diminished with favorable outcome from the percutaneous drainage. The solution to resolving this complication is early diagnosis and percutaneous drainage under CT guidance. The current body of knowledge is still small, and further research is required to recognize risk factors and prevent future biloma occurrence, reducing morbidity and mortality of the patients.

## 4 Conclusion

Post laparoscopic biloma cysts are a very rare case. Management with percutaneous drainage in an outpatient clinic and ambulatory drainage is an effective and safe procedure.

## Ethical approval

The informed consent form was declared that patient data or samples will be used for educational or research purposes. Our institutional review board also do not provide an ethical approval in the form of case report.

## Sources of funding

The authors declare that this study had no funding source.

## Author contribution

Adeodatus Yuda Handaya conceived the study and critically revised the manuscript for important intellectual content. Aditya Rifqi Fauzi, Ahmad Shafa Hanif, Joshua Andrew, Kevin Radinal, and Azriel Farrel Kresna Aditya drafted the manuscript. Adeodatus Yuda Handaya, Aditya Rifqi Fauzi, Ahmad Shafa Hanif, Joshua Andrew, Kevin Radinal, and Azriel Farrel Kresna Aditya facilitated all project-related tasks.

## Research registration number

The manuscript is a case report, not considered a formal research involving participants.

## Guarantor

Adeodatus Yuda Handaya.

## Provenance and peer review

Not commissioned, externally peer-reviewed.

## Declaration of competing interest

No potential conflict of interest relevant to this article was reported.
